# Performance of Mortars with Commercially-Available Reactive Magnesium Oxide as Alternative Binder

**DOI:** 10.3390/ma14040938

**Published:** 2021-02-16

**Authors:** Miguel Bravo, Javier A. Forero, José Nobre, Jorge de Brito, Luís Evangelista

**Affiliations:** 1 CERIS, Escola Superior de Tecnologia do Barreiro, Instituto Politécnico de Setúbal, Rua Américo da Silva Marinho, 2839-001 Lavradio, Portugal; 2 Postgraduate Program in Structural Engineering and Construction (PECC), Prédio SG-12 Campus Darcy Ribeiro, University of Brasília, Brasilia-DF CEP 70910-900, Brazil; javier.valencia@tecnico.ulisboa.pt; 3 Instituto Superior Técnico, Universidade de Lisboa, Av. Rovisco Pais, 1049-001 Lisboa, Portugal; jose.nunes.nobre@ist.utl.pt; 4 CERIS, Instituto Superior Técnico, Universidade de Lisboa, Av. Rovisco Pais, 1049-001 Lisboa, Portugal; jb@civil.ist.utl.pt; 5 CERIS, Instituto Superior de Engenharia de Lisboa, R. Conselheiro Emídio Navarro, 1950-062 Lisboa, Portugal; evangelista@dec.isel.ipl.pt

**Keywords:** MgO, reactivity, mechanical properties, durability properties, shrinkage, alternative binder

## Abstract

This paper intends to analyze the performance of mortars with reactive MgO, as a sustainable alternative to cement. Six different MgOs from Australia, Canada, and Spain were used in the production of mortars as partial substitutes for cement, namely 5%, 10%, 15%, 20%, and 25% (by weight). MgOs with different levels of reactivity were used to analyze its influence on the performance of MgO mortars. In order to evaluate the mechanical performance of these mortars, compressive strength, flexural strength, dynamic modulus of elasticity, and ultrasonic pulse velocity tests were performed. Compressive strength tests showed that the use of 25% reactive MgO can cause a decrease of this property of between 28% and 49%. The use of reactive MgO affected the other mechanical properties less. This paper also intends to analyze the durability performance of mortars with reactive MgO. To that effect, water absorption by capillarity was assessed. In this research, the effect of using MgO on the shrinkage was also analyzed. It was found that shrinkage may decrease by more than a half in some cases.

## 1. Introduction

The world population is estimated to reach 9.7 billion in 2050 and 10.9 billion in 2100. Population growth directly affects the construction sector, as this sector is responsible for providing the necessary housing conditions through the development of infrastructure [1,2,3]. Currently, the construction sector is responsible for the emission of 11% of the total carbon dioxide (CO_2_) expelled to the atmosphere per year [4], with the cement industry emitting approximately 5.7 billion tons of CO_2_ in 2018 [5]. Therefore, one of the alternatives to mitigate this problem in the construction sector is the incorporation, to the detriment of ordinary Portland cement, of sustainable materials such as fly ash (FA), active silica, slag, metakaolin, reactive MgO, among others.

As mentioned, an alternative to reduce the CO_2_ emission caused by the Portland cement production process is the use of MgO in the production of new concrete. This reactive MgO can be produced by the calcination of magnesite (MgCO_3_) at much lower temperatures (700 to 1000 °C) than those used in the production of Portland cement [6]. In addition, concrete produced with MgO can allow permanent sequestration of CO_2_ over its lifetime, thus partly offsetting the CO_2_ emitted in its production phase [7]. As well as this environmental advantage, concrete with reactive MgO may behave better in some aspects, e.g., in terms of shrinkage [8].

The physical and chemical properties of MgO are directly related to the process of calcination of magnesite (MgCO_3_ → MgO + CO_2_). Thus, the MgO obtained by magnesite calcination is currently divided into four types, depending on the calcination temperature used in the process, which can vary between 800 °C and 2800 °C. The first type of MgO is called light-burned or caustic-calcined and results from the process of calcining magnesite at temperatures between 700 °C and 1000 °C. In this process, a MgO (purity) content of more than 85% is obtained, and it presents a higher reactivity and a larger specific surface area. MgO is called hard-burned MgO when it is calcined at temperatures ranging from 1000 °C to 1500 °C and has a lower reactive degree and specific surface than the previous one. MgO is designated dead-burned MgO or periclase when it is produced at temperatures between 1400 °C and 2000 °C. Finally, MgO is called fused MgO when it is calcined at 2800 °C, producing an MgO with practically zero reactivity that is mainly used in the steel industry [9,10].

MgO additions to cementitious materials can be made mainly through two methods. The first is to add an MgO quantity to the clinker, producing high magnesium cements. These are used in refractory concrete (Al_2_O_3_–MgO system), where the reaction of magnesia with alumina (from 1000 °C) results in the formation of spinel in situ, which has a higher resistance to penetration by slag than equivalent compositions containing preformed spinel [11,12]. The second method consists of preparing MgO from the calcination of magnesite (MgCO_3_) and then incorporating MgO into concrete as a binder [11]. The use of MgO as a binder results in the formation of Mg(OH)_2_ (Equation (1)) and its subsequent carbonation (Equation (2)), giving rise to hydrated magnesium carbonates. This type of binder was designed to replace Portland cement in large quantities, obtaining environmental benefits in relation to CO_2_ emissions [13].

MgO + H_2_O → Mg(OH)_2_ (1)

Mg(OH)_2_+ CO_2_ + 2H_2_0 → MgCO_3_·3H_2_O (2)

Research carried out by several authors on the incorporation of different reactive MgO contents (with low calcination temperatures) in cement materials has shown that there is a reduction in mechanical properties, such as compressive strength [14,15,16,17,18,19,20,21,22], flexural strength [17,18,21,23,24], and tensile strength [25].

Liu et al. [14] showed that there is a reduction of up to 15% in mixes produced with 3% MgO contents tested at different ages. Mo et al. [18] tested mortars with 8% MgO incorporation with different degrees of reactivity (reactivity values of 50 s, 100 s, 200 s, and 400 s), observing a reduction of approximately 18% in compressive strength at 28 days for the least reactive MgO. This decrease was more marked at advanced ages (14% at 91 days). The authors also observed that the degree of reactivity of MgO did not influence the strength of the mortars.

In another study by Mo et al. [16], where FA and MgO were incorporated in mortars exposed to pressures of 0.55 MPa and 0.10 MPa of CO_2_ for 3 and 24 h, it was observed that mortars exposed to a higher amount of CO_2_ had an increase in strength between 7% and 72% compared to mortars exposed to current pressures.

Regarding flexural strength, Gonçalves et al. [26] obtained decreases of between 27% and 30% in mortars, with MgO contents of up to 20%. In turn, Moradpour et al. [23] studied the influence on flexural strength of the incorporation of nano-MgO in mortars at various contents (up to 5%) and carried out the test at different ages (between 7 days and 90 days). The authors obtained an increase in flexural strength in mortars with nano-MgO, regardless of the age of the test and the content of nano-MgO used. However, it should be noted that these results may be due to a greater compactness of these mortars due to a filler effect. The use of other types of additions in conjunction with reactive MgO has shown positive results, reaching values similar to those of conventional mortars. This was also observed by Wei et al. [27], who produced mortars with different contents of microsilica and MgO and with no ordinary Portland cement, observing results similar to conventional mortars, when the proportion of the mix was 30% microsilica and 70% reactive MgO.

The modulus of elasticity is also influenced by the incorporation of MgO. Gonçalves et al. [26] studied the modulus of elasticity in mortars with reactive MgO, obtaining a slight reduction (between 9% and 15%) with the use of different types of MgO. The authors justify this reduction with the higher water content of mortars with MgO, which is necessary to maintain the same consistency in all mortars.

Regarding the durability behavior of cementitious materials with addition of MgO, Mavroulidou et al. [20] evaluated the influence of the use of 5% and 10% MgO on the water absorption in mixes with FA and metakaolin. The authors found that the incorporation of 5% MgO decreased water absorption, which was attributed to the better compaction of mixes with higher levels of MgO and metakaolin, due to the greater water requirements in its composition to maintain consistency. With the incorporation of 10% MgO, the authors obtained better results than those of the reference mortars, but worse than those for mortars with 5% MgO.

Moradpour et al. [23] found that the permeability of mortars decreases (7% to 33%) with the use of nano-MgO. According to Dung and Unluer [28], the permeability of cementitious materials with MgO can decrease further (up to 24%) if HCI (hydration agent (HA)) or NaHMP (dispersion agent (DA)) is incorporated simultaneously.

As is well known, CO_2_ absorption is higher in MgO cementitious materials, increasing their carbonation. Pu and Unluer [25] analyzed the degree of carbonation at 14 days of concrete blocks made with an MgO content of 0% to 10%. The authors found that the degree of carbonation of concrete with MgO is twice that of the reference mix. This increase was also reported by Gonçalves et al. [26], who found that the carbonation depth at 28 days in mortars with 20% MgO increased by between 139% and 483%, depending on the reactive MgO used.

The use of MgO in cementitious mixes greatly affects their shrinkage. This positive aspect is attributed to the fact that the hydration of MgO leads to compensation of shrinkage, as described by Mo et al. [17]. Kabir and Hooton [29] observed a reduction of shrinkage by more than 50% in concrete with 15% reactive MgO content. The authors also found that the use of low reactivity MgO did not cause the same effect and obtained shrinkage similar to that of the reference concrete. Jin et al. [27] also concluded that the influence on shrinkage of the use of MgO depends on the degree of reactivity of the MgO used, having obtained maximum decreases of 26% when using a highly reactive MgO.

This paper presents the experimental results in mortars incorporating six different types of reactive MgO obtained from three manufacturers located in Spain, Australia, and Canada. So far there is not such a comprehensive study in the literature where it is possible to compare the performance in mortars of as many different types of MgO. This extensive analysis is especially important in order to understand the behavior of mortars with reactive MgO, as well as the reasons for it. In other words, by analyzing the incorporation of six different reactive MgOs (with different reactivity and calcination temperatures), it is possible to understand the influence that the variation of the properties of MgO has on the mixes produced with these MgO. On the other hand, in this research, cement substitution contents of 0%, 5%, 10%, 15%, 20%, and 25% were used in order to evaluate whether the behavior of mortars with MgO shows a linear behavior versus the substitution content, or whether, on the contrary, there is a change in trend from a given substitution ratio. All the mortars produced were evaluated in mechanical and durability terms. To determine the mechanical performance of mortars with MgO, tests of compressive strength, flexural strength, dynamic modulus of elasticity, and ultrasonic pulse were performed. In turn, the capillarity water absorption test was carried out to evaluate the durability of the mortars. In addition to these tests, the shrinkage of the mortars was evaluated over 91 days. Thus, it was possible to carry out a fairly complete characterization of the mortars with reactive MgO. This extension of the experimental campaign will make it possible to carry out a global analysis that is not yet available in the literature. Currently, there is still no extensive experimental campaign in the literature that allows an accurate evaluation of the influence of the incorporation of MgO as a binder in cementitious materials. On the other hand, the few existing investigations do not use MgO with different characteristics (reactivity), in order to understand how its reactivity affects the properties of cementitious materials.

## 2. Experimental Program

### 2.1. Tests

The experimental campaign was divided into three phases. In the first one, the various materials used in the production of the mortars were analyzed. Subsequently, in the second and third phases, the produced mortars were tested in their respective fresh and hardened states.

To determine the properties in the fresh state of the mortars, consistency and density tests were carried out in accordance with the standards EN 1015-3 [30] and EN 1015-6 [31], respectively. On the other hand, to determine the mechanical characteristics of mortars, compressive and flexural strength, ultrasonic pulse, and dynamic modulus of elasticity tests were carried out following standards EN 1015-11:1999/A1 [32], EN 15,317 [33], and ASTM E1876-15 [34], respectively. The compressive and flexural strengths were analyzed at 7, 28, and 91 days, using 6 and 3 specimens, respectively. The ultrasonic pulse and dynamic modulus of elasticity tests were performed at 28 days in 3 specimens.

In order to characterize the durability performance of the mortars, the water absorption by capillarity was determined in accordance with standard EN 1015-18 [35]. This test was performed at 28 days in 6 specimens.

Finally, the shrinkage of the mortars over a period of 91 days was evaluated in accordance with standard EN 1015-13 [36], using 3 specimens.

### 2.2. Materials

The cement used as the main binder in mortar compositions was CEM I 42.5 R, which was produced by Secil (Setúbal, Portugal). Two siliceous sands with grain sizes of 0–2 mm and 0–4 mm were also used, fulfilling the requirements of EN 12,620 [37]. Six types of MgO from three different manufacturers located in Spain, Australia, and Canada were used to investigate the influence of the use of MgO in mortars. MgO from Spain was designated S0, S1, and S2, depending on the milling process and consequently the size of the MgO. The MgO designated S0 corresponds to MgO not yet subjected to any grinding process after it was supplied by the Spanish company. In turn, when this material was subjected to a milling treatment in a rotating ball mill for one and two hours, the resulting MgOs were designated MgO S1 and MgO S2, respectively. The grinding was done with different diameters of steel balls (2 cm and 4 cm) and aimed at obtaining a MgO with a size distribution similar to that of cement. Two types of MgO from Canada were used and designated C30 and C40. These MgOs supplied by the manufacturer differ in terms of size distribution and reactivity. Finally, only one type of MgO from Australia was used, and was designated MgO-A. Canada’s and Australia’s MgOs were not treated because they already had size ranges similar to that of cement. Only the Spanish MgO was subjected to a treatment in order to obtain sizes similar to that of cement. Thus, it is also possible to check the importance of the MgO size on its application in cementitious materials.

Table 1 shows the results obtained in the tests to determine the specific surface and purity of the several MgO analyzed. The specific surface affects the reactivity of MgO and is therefore an extremely important element. In turn, the purity of MgO makes it possible to determine how many other elements there are in the six different MgO received from the manufacturers. This factor also significantly influences the behavior of the mortars produced with these materials.

From Table 1, it can be concluded that MgO-C30, MgO-C40, and MgO-A have reactivity and specific surfaces much higher than those of cement and other MgOs. Liska et al. [38] also showed that the reactivity of MgO increases by reducing its particle size and, consequently, increasing its specific surface area.

It can also be seen that the product from Australia has the highest content of MgO in its constitution (98.8%). All MgOs have a purity higher than 85%.

### 2.3. Mix Design

**Figure 1:**
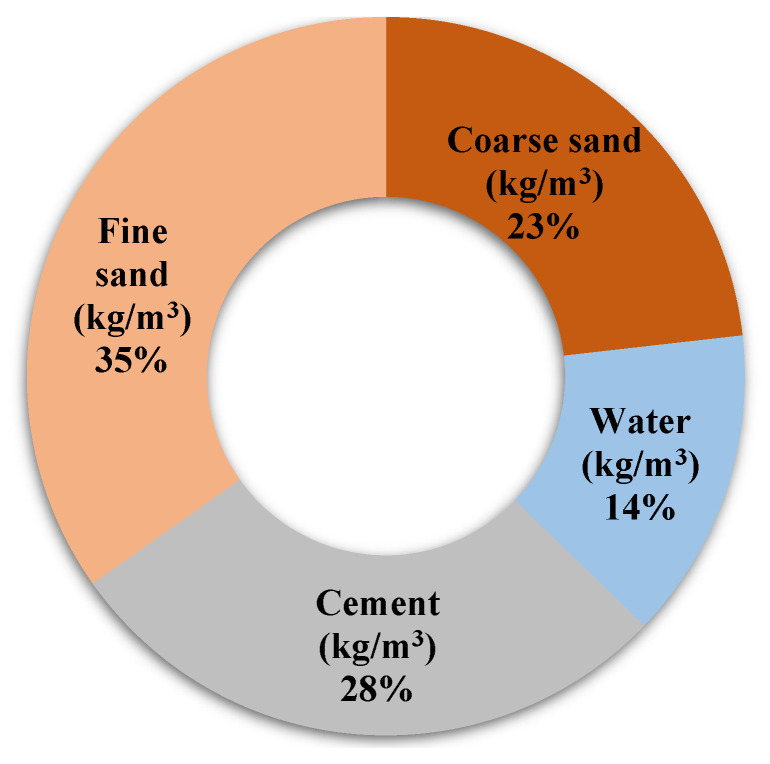
Composition of the reference mortar (RM) (kg/m^3^).

To determine the composition of each mix, the methodology proposed by Nepomuceno et al. [39] and a water/binder (w/b) ratio of 0.50 were used. The standard composition of the mixes can be seen in Figure 1. Cement substitutions with MgO were made by mass: 0%, 5%, 10%, 15%, 20%, and 25%. The designation of each mix is composed of the percentage of MgO and the origin of the MgO. For example, the reference mix was designated M0, and mixes with MgO from Australia were designated M5:A, M10:A, M15:A, M20:A, and M25:A. In total, 31 mortars mixes were produced.

**Figure 2:**
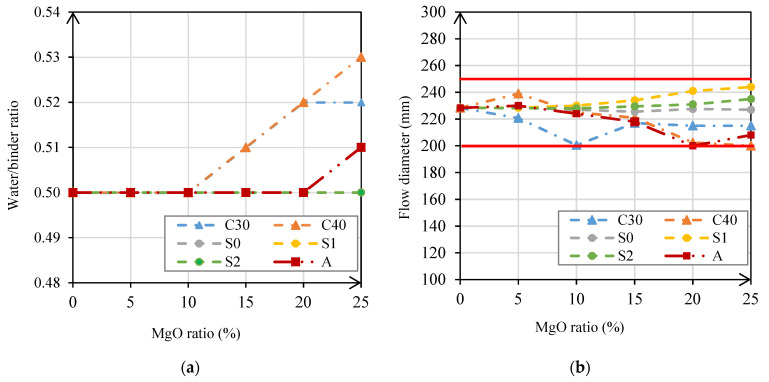
W/b ratio of each mix (**a**); mortar consistency according to the MgO content (**b**).

This experimental campaign maintained the consistency of the mortars between 200 mm and 250 mm of flow diameter, which in some specific cases implied an increase in the w/b ratio and, consequently, in the amount of water used in the mortars. The w/b ratio values are presented in Figure 2a, as well as the results obtained for consistency (Figure 2b).

Figure 2a shows that maintaining the consistency of mortars with MgO-S0, MgO-S1, and MgO-S2 did not imply an increase in the w/b ratio of these mixes. In mortars produced with MgO-C30, MgO-C40, and MgO-A, the flow diameter decreased with the increase in MgO content. Therefore, it was necessary to increase the w/b ratio with the increase in the ratio of substitution of the binder in order to maintain consistency in the target range. As shown in Table 1, these three types of MgO have a much larger specific surface area than the others. Thus, in mortars with these types of MgO, the greater angularity and roughness of the MgO particles may have caused an increase in the amount of water adsorbed and, consequently, a decrease in the flow diameter of mortars.

## 3. Results and Discussion

### 3.1. Fresh Mortar Properties

**Figure 3:**
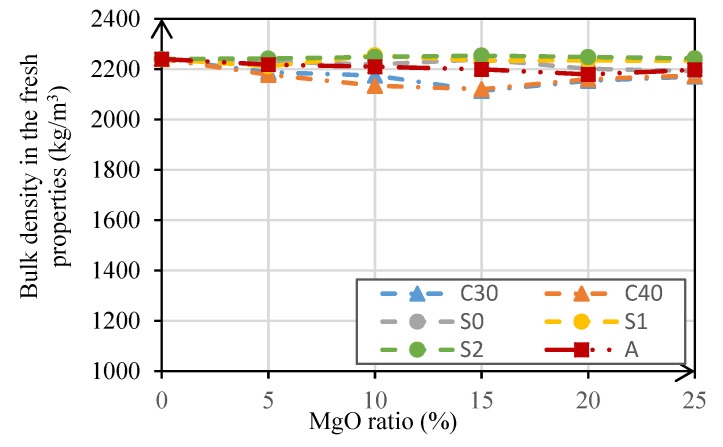
Mortar bulk density as a function of the MgO content.

The results of the bulk density test presented in Figure 3 show that this property always remained between 2114 kg/m^3^ and 2255 kg/m^3^. For mortars with MgO-S0, MgO-S1, MgO-S2, and MgO-A, bulk density was approximately constant between 2179.2 kg/m^3^ and 2254.8 kg/m^3^. For mortars produced with MgO-C30 and MgO-C40, the bulk density was slightly reduced in relation to the reference mortar (between 2.3% and 5.6%). This decrease may have been caused by an increase in the w/b ratio. Recall that only the mortars produced with these two types of MgO are needed to increase the w/b ratio in order to maintain constant consistency.

### 3.2. Hardened State Properties

#### 3.2.1. Compressive Strength

**Figure 4:**
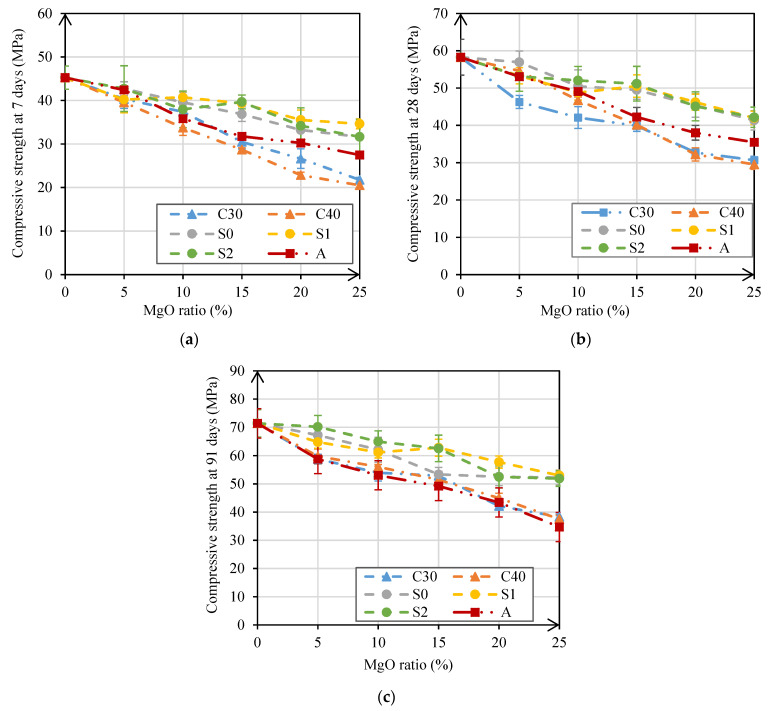
Compressive strength at 7 days (**a**); 28 days (**b**); and 91 days versus MgO content (**c**).

The compressive strength test is one of the most important to characterize the mechanical performance of mortars. The compressive strength results at 7, 28, and 91 days are shown in Figure 4a–c. As the results were obtained from the analysis of several specimens, the standard deviation of each of the results was also presented in the figure. A high standard deviation naturally means that the data are widely spread, and a low standard deviation indicates that all data are close to the average value.

As expected, with the increase in MgO content, the compressive strength decreased in all mortars and at all ages. According to Mo et al. [18], this reduction is due to the decrease in the amount of cement used in mortars, since magnesium oxide hydration products are less resistant than those of cement. Therefore, the greater the amount of MgO used as partial replacement of cement, the greater the reduction in strength of the mortars.

The mortars most affected by the incorporation of MgO were those made with MgO-C30 and MgO-C40, with reductions of more than 40% at 28 days, when 25% of MgO was used. This negative influence of these MgOs may be because these mortars needed an increase in the water/binder ratio. On the contrary, the mortars least affected were those produced with MgO-S0, MgO-S1, and MgO-S2. In these cases, the use of 25% of these MgOs caused maximum reductions of 30% at all ages.

The mortars produced with MgOs from Spain (S0, S1, and S2) showed very similar performances, although they had different size distributions. These results indicated that the size of the MgO used does not significantly affect the mortars in terms of compressive strength.

The best results obtained in mortars with MgO from Spain may be due to the high presence of SiO_2_ (>5.8%) in the composition of these MgOs, contrary to the MgOs from Canada and Australia (<0.4%). The presence of SiO_2_ leads to pozzolanic reactions with Mg(OH)_2_, which promote further development of the strength of the cementitious paste [19]. Another factor that may have caused a greater reduction in mechanical strength in mortars with MgOs from Canada is their higher reactivity. This may have led to an initial expansion in these mortars that may have resulted in the appearance of microcracking. Moreover, the high reactivity of the MgOs from Canada may have led to the initial formation of Mg(OH)_2_ that, by precipitating in the anhydrous cement grain environment, may have hindered the hydration of the cement grains.

**Figure 5:**
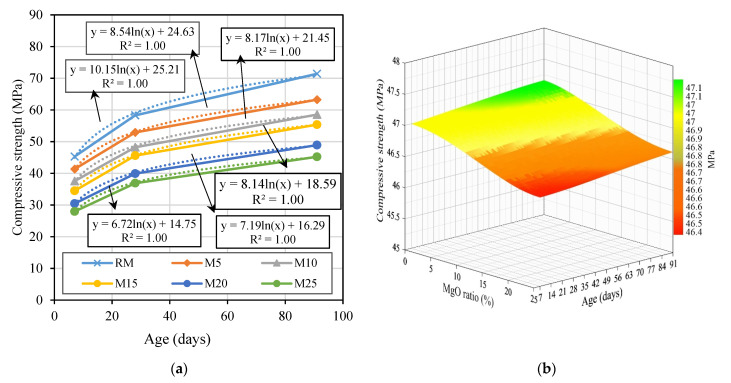
Compressive strength over time of mortars with MgO (**a**); compressive strength over time versus incorporation ratio of MgO (**b**).

Figure 5 shows that the evolution of compressive strength in mortars with reactive MgO was identical to that of the reference mortar. The compressive strength at 7 days of RM was 11%, 18%, 34%, 32%, and 38% higher than the average values of compositions M5, M10, M15, M20, and M25, respectively. In turn, the compressive strength at 91 days decreased by 11%, 16%, 26%, 27%, and 36% with the addition of 5%, 10%, 15%, 20%, and 25% reactive MgO, respectively. As shown in Figure 5, these values indicated that the evolution of compressive strength was identical in mortars with Portland cement and in those with reactive MgO. However, it should be noted that the reference mortar was produced with CEM I 42.5 R, which caused a high initial strength. Therefore, it was proven that the reactive MgO also allows high strengths to be obtained in the first ages of the mortars. It was also found that the evolution of compressive strength of all mortars followed a logarithmic relationship, with coefficients R^2^ equal to 1.0 at the three ages (Figure 5a).

#### 3.2.2. Flexural Strength

**Figure 6:**
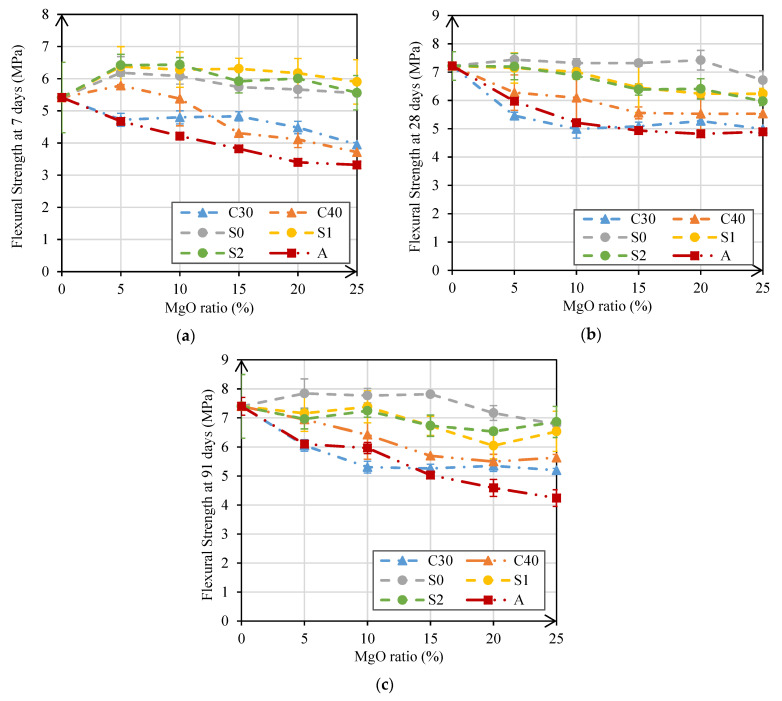
Flexural strength at 7 days (**a**); 28 days (**b**); and 91 days (**c**) versus MgO content.

The results obtained in the flexural strength test at 7 days, 28 days, and 91 days are presented in Figure 6a–c. At 7 days, the results can be divided into two trends. In mortars produced with MgO-S0, MgO-S1, and MgO-S2, there was an increase in flexural strength between 12% and 19% for MgO ratios up to 10%. On the other hand, for higher replacement ratios, values close to those observed for the reference mortar were obtained. The increases observed in the flexural strength in mortars with MgO were not expected, since the incorporation of MgO implies a reduction in the amount of cement and should therefore lead to a reduction in flexural strength, as for compressive strength. However, it is possible that the hydration products formed in the mortars with MgO from Spain led to an improvement of the ITZ between the aggregates and the paste, which, as is well known, is fundamental for flexural strength. However, this hypothesis needs to be proven by scanning electron microscopy (SEM) tests. In the case of mortars produced with MgO from Canada and Australia, maximum decreases between 27% and 39% were observed when 25% cement was replaced.

Figure 6b,c shows that, at 28 days and 91 days, the flexural strength of mortars with MgO maintained the same decreasing trend. It should be noted that in mortars with MgO-C30 and MgO-A, the greatest decreases were achieved (in the order of 30%), while in mortars with MgO-S0 the smallest decreases occurred, maintaining approximately the strength found in the reference mortar. For the reasons explained in the compressive strength analysis, it was considered that the best results obtained in mortars with MgOs from Spain may have been due to the high presence of SiO_2_ (>5.8%) in the composition of these MgOs and the lower reactivity, compared with the others. Wei et al. [27] also studied mortars with different contents of microsilica and MgO and with no ordinary Portland cement, observing good results (similar to conventional mortars).

**Figure 7:**
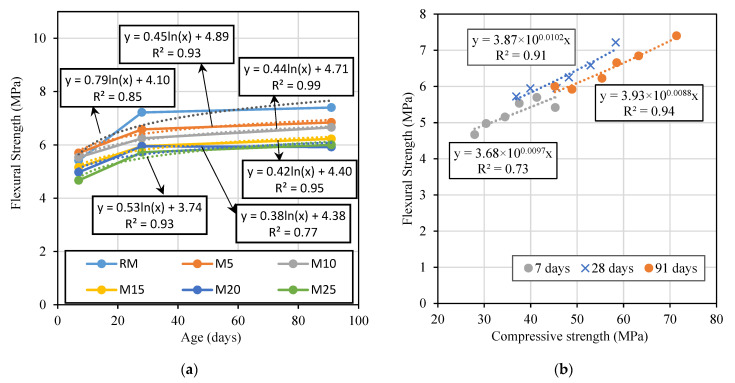
Flexural strength over time in MgO mortars (**a**); relationship between compressive and flexural strength (**b**).

Through Figure 7a, it is possible to better understand the evolution of flexural strength over time. There, it can be seen that the evolution of this property over time in mortars with MgO was quite distinct from that of the reference mortar. There seemed to be a clear trend for increased flexural strength in mortars with MgO, mostly until 7 days of age. On the contrary, in the reference mortar, there was a 33.3% increase in flexural strength from 7 days to 28 days of age. These results prove that the use of reactive MgO causes a faster occurrence of hydration reactions in mortars.

Figure 7b shows that there was a good correlation between compressive strength and flexural strength. The best correlations between these two characteristics occurred in the tests at 28 days and 91 days, with R^2^ of 0.91 and 0.94, respectively. On the contrary, the correlation between these properties at 7 days was not so clear (R^2^ of 0.73). This may be explained because the rapid hydration of MgO caused rapid increases in flexural strength of these mortars due to the fast strengthening of ITZ between the aggregates and the paste. In the case of compressive strength, this better initial behavior of mortars with MgO was not so visible. It was also found that the evolution of flexural strength of all mortars followed a logarithmic relationship, with coefficients R^2^ higher than 0.77 at the three ages (Figure 7a).

#### 3.2.3. Dynamic Modulus of Elasticity

**Figure 8:**
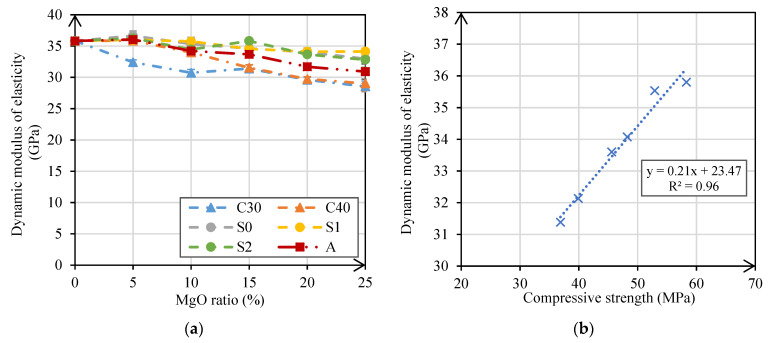
Modulus of elasticity versus MgO content (**a**); compressive strength versus modulus of elasticity (**b**).

The modulus of elasticity was determined by an indirect process, using the resonance frequency of the mortar specimen and specific software. The results of the 28-day dynamic modulus of elasticity test are shown in Figure 8. They show a slight decrease of this property with an increase in the content of MgO in all mortars. Gonçalves et al. [26] evaluated mortars with different types of MgO and also obtained slight reductions (less than 10%) of this property with the incorporation of 20% MgO.

In mortars made with MgO-S0, MgO-S1, and MgO-S2, maximum decreases of 8%, 5%, and 8%, respectively, were achieved. In these mixes, it was again found that grinding time had no influence on the modulus of elasticity, and approximately equal results were obtained in the three mortars made with MgO from Spain. The greatest variation occurred in the mortars produced with C30 and C40, which had a maximum reduction of 20% in relation to the reference mortar. In mortars produced with MgO-A, this property was reduced by 14% at the replacement ratio of 25%. The increase in water/binder ratio is the main reason for the decrease in dynamic modulus of elasticity of the mortars with these three types of MgO. Gonçalves et al. [26] also concluded that the decrease in this property is most likely due to the need to add more water to obtain equivalent workability levels.

In Figure 8b, as suggested by Yedra et al. [40], it is possible to observe the relationship between the compressive strength and the dynamic modulus of elasticity of the mortars produced in this research. The linear correlation between compressive strength and dynamic modulus of elasticity was quite good, with an R^2^ of 0.96.

#### 3.2.4. Ultrasonic Pulse Velocity

**Figure 9:**
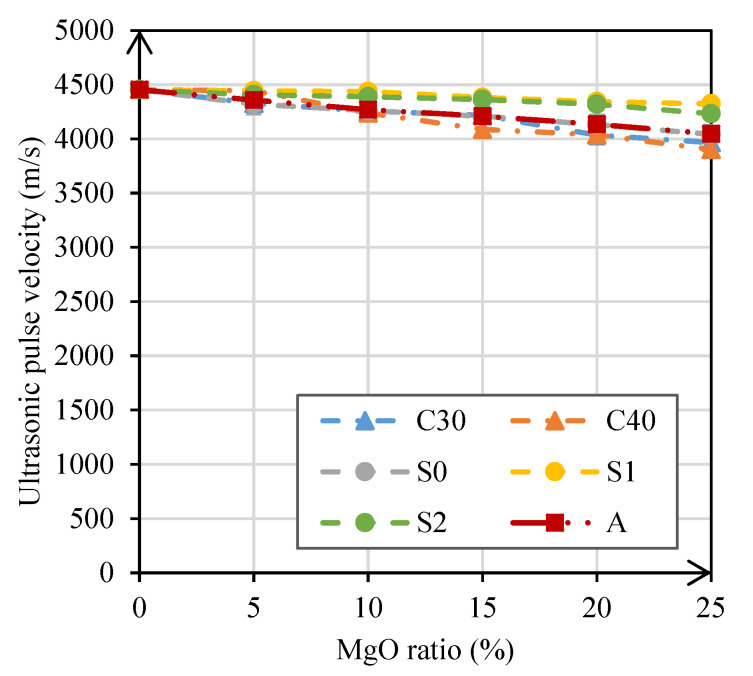
Ultrasonic pulse velocity versus MgO content.

The results of the ultrasonic pulse velocity test at 28 days are shown in Figure 9, which shows that the propagation of ultrasonic pulse slowed down with an increase in the content of MgO. These results indicate that the use of MgO led to a more porous mortar. This variation could be either a consequence of an initial expansive cracking or of the different hydration processes of the binders. Lafhaj et al. [41] observed that reductions in this property tend to be directly related to increases in porosity or water content of the mortars.

In mortars produced with MgO-S1 and MgO-S2, maximum reductions in ultrasonic pulse velocity of only 3% and 5%, respectively, were obtained. It should be noted that, relative to this test, more favorable results were obtained in mortars with MgO-S1 and MgO-S2 than in those with MgO-S0, which means that the grinding of MgO from Spain was beneficial for this property. The higher porosity of the mortars with MgO-S0 can be proved in the analysis of water absorption by capillarity.

In mortars with MgO-C30, MgO-C40, and MgO-A, similar results were obtained, with maximum reductions of 11%, 12%, and 12%, respectively. Thus, the MgOs that caused the greatest decrease in the density of mortars (Canadian and Australian) were also the ones that caused the greatest decrease in ultrasonic pulse velocity. These results prove the strong influence that the density of cement materials has on the ultrasonic pulse velocity.

#### 3.2.5. Water Absorption by Capillarity

**Figure 10:**
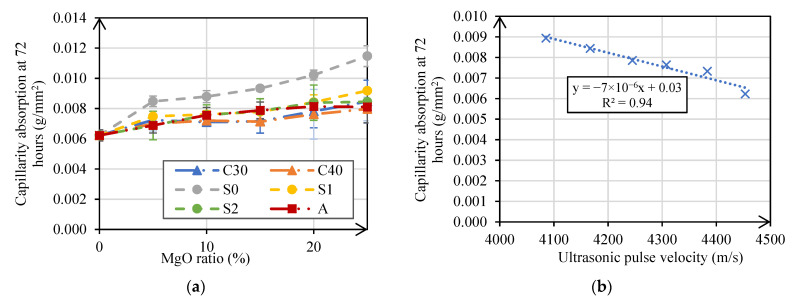
Water absorption by capillarity at 72 h versus MgO content (**a**); ultrasonic pulse velocity versus water absorption by capillarity (**b**).

The results of the absorption of water by capillarity test at 28 days are presented in Figure 10. The absorption of water by capillarity increased with the increase in MgO content. The use of 5%, 10%, 15%, 20%, and 25% of MgO in mortars resulted, on average, in an increase of water absorption by capillarity, in comparison with the reference mortar, by 18%, 23%, 26%, 36%, and 44%, respectively.

The worst results obtained in mortars with MgO-S0 are particularly noteworthy. They show that, although the MgO present in these mortars formed hydration products of good strength, as the results of the mechanical tests performed on the mortars proved, the large size (low specific surface) of MgO-S0, in comparison with cement and the other MgOs, may have originated the production of mortars with high porosity. This aspect can be proved by the results from the ultrasonic pulse velocity test, since porosity is also directly linked to the transmission capacity of ultrasonic waves [42]. Mo and Panesar [43] observed that the total pore volume of cement pastes made with MgO was up to 10% higher than that of the reference paste. Figure 10b shows the relationship between water absorption by capillarity and ultrasonic pulse velocity, with a linear correlation an with R^2^ of 0.94. Zhang and Panesar [44] observed increases in water absorption when analyzing cementitious materials containing reactive MgO up to 90%. They found that this result was due to an increase in the quantity and pore size of these cementitious materials.

#### 3.2.6. Shrinkage

**Figure 11:**
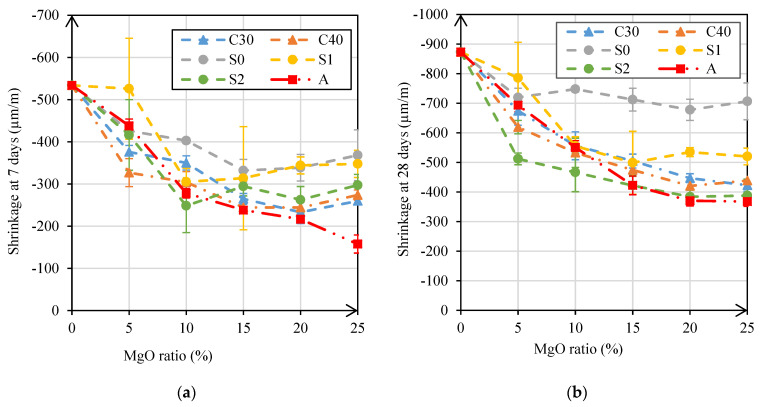

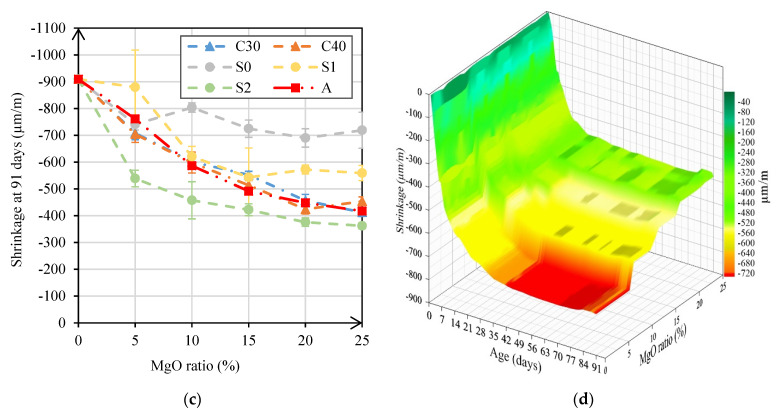
Shrinkage at 7 days (**a**), 28 days (**b**), and 91 days (**c**) versus MgO content; shrinkage over time versus incorporation ratio of MgO (**d**).

The shrinkage test is very important to characterize the durability of a mortar, especially in this study where the incorporation of MgO can lead to important variations in this property, namely a reduction in shrinkage or even the occurrence of initial expansions. The variation of shrinkage at 7, 28, and 91 days with the increase in MgO content is represented in Figure 11a–c, respectively.

Figure 11c shows that the shrinkage significantly decreased with the increase in MgO content. This is in line with expectations, as this is exactly one of the advantages of using MgO as a substitute of cement. The shrinkage reduction was mostly up to the 15% substitution ratio, after which shrinkage remained constant.

The mortars produced with MgO-S0 were those in which there was a smaller reduction in shrinkage at 91 days, with a maximum decrease of 24%. This result may be due to the larger particle size of this MgO, compared to the others. Thus, it can be seen that, relative to the shrinkage test, grinding of the Spanish MgO led to a significant difference in results. This is because a decrease in size of the MgO caused an increase in the expansive hydration reactions with MgO. Mo et al. [18] also observed that shrinkage significantly decreased with the use of MgO due to the hydration reactions of MgO.

In the mortars with MgO-C30, MgO-C40, MgO-A, and MgO-S2, the greatest reductions in shrinkage were achieved, with decreases of more than 50%. In this case, the production of mortars with more reactive MgOs caused a more significant reduction in shrinkage.

In Figure 11d, the evolution of shrinkage over time can be observed as a function of the binder replacement ratio. In this figure, it can be clearly seen how the substitution of cement with reactive MgO improves the shrinkage of the mortars.

## 4. Conclusions

This research has served to assess the influence of the substitution of cement with reactive MgO in mortars. Table 2 presents a summary of the results obtained for the reference mortar (RM) in the several tests performed, as well as the variations resulting from the use of 5%, 15%, and 25% of the reactive MgOs analyzed. In order to more easily understand the results obtained, those variations have different codes depending on the results obtained. Thus, positive variations are marked with (+), negative variations below the ratio of substitution of cement with MgO are marked with (-), and negative variations above the ratio of substitution are marked with (--).

In summary, after analyzing the fresh and hardened performance of the mortars with MgO of different origins, some conclusions are drawn. From the analysis of the results obtained in the fresh state, both for density and consistency, it is concluded that the incorporation of MgO did not lead to significant variations of these properties, keeping the values obtained within the target ranges.

In the compressive strength test, the reductions exceeded 45% for mortars with 25% of MgO-C30 and MgO-C40. The best performance was obtained in mortars produced with MgO from Spain, where maximum reductions of 30% were achieved.

In the 28-day flexural strength test, maximum reductions of about 30% were observed in mortars produced with 25% MgO-C30, MgO-C40, and MgO-A. The best performances in this property were obtained in mortars with MgO from Spain, where similar results to those of the reference mortar were obtained. It should be noted that at 7 days of age, most of the mortars produced with these MgO had the same flexural strength as the reference mortar.

The modulus of elasticity and ultrasonic tests also showed a loss of performance, albeit reduced, when incorporating MgO in mortars. In the modulus of elasticity test, the maximum reductions were between 5% and 20%, corresponding to mortars with MgO-S1 and MgO-C30, respectively. In the ultrasonic pulse velocity test, maximum reductions between 3% (mortars with MgO-S1) and 12% (mortars with MgO-C40) were observed. It should be noted that despite the qualitative decreases observed in these tests when using MgO, there was almost always a decrease in the results below the ratio of substitution of cement.

In relation to the water absorption by capillary test, performance losses were higher. Absorption values were observed in mortars produced with MgO-S0 up to 84% higher than the results obtained in the reference mortar. However, in mortars with MgO-C40 and MgO-A, maximum reductions of less than 30% were obtained.

In the shrinkage test, the use of MgO-C30, MgO-C40, MgO-A, and MgO-S2 reduced the shrinkage of mortars by more than 50%. By contrast, the use of MgO-E0 only allowed the shrinkage of mortars to be reduced by about 21%. It was also possible to conclude that the significant increase in the reduction of shrinkage, with an increase of the ratio of MgO, occurred mostly up to a ratio of 15%, after which the shrinkage remained approximately constant.

The treatment (grinding) of MgO from Spain significantly improved the behavior of mortars produced with this MgO, both in terms of water absorption by capillarity and shrinkage. This was not the case in the mechanical tests. It can therefore be concluded that the latter depend more on the composition of the MgO used in the mortars than on their size distribution.

## Figures and Tables

**Table 1:** Characteristics of the various reactive MgO and cement.

Binder	MgO-S0	MgO-S1	MgO-S2	MgO-C30	MgO-C40	MgO-A	Cement
Purity (%)	85.0	86.3	86.2	95.9	96.0	98.8	-
Specific surface (m^2^/g)	4.9	3.0	2.6	28.6	47.9	51.2	2.8
Reactivity (s)	3544	1460	1777	21	16	14	-

**Table 2:** Summary of mechanical and durability properties of mortars with cement substitution with MgO.

Properties of Mortars	Compressive Strength at 28 Days	Flexural Strength at 28 Days	Dynamic Modulus of Elasticity	Ultrasonic Pulse Velocity	Water Absorption by Capillarity	Shrinkage at 91 Days
RM	58.3 MPa	7.2 MPa	35.8 GPa	4453.3 m/s	6.22 × 10^−3^ g/mm^2^	−909.3 µm/m
M5-A	−17% (--)	−17% (--)	−1% (-)	−2% (-)	+11% (--)	−16% (+)
M5-C30	−21% (--)	−24% (--)	−14% (--)	−3% (-)	+16% (--)	−22% (+)
M5-C40	−6% (--)	−13% (--)	0%	0%	+12% (--)	−23% (+)
M5-S0	−2% (-)	+3% (+)	−2% (-)	−3% (-)	+36% (--)	−19% (+)
M5-S1	−9% (--)	−1% (-)	0%	0%	+20% (--)	−3% (+)
M5-S2	−9% (--)	0%	−1% (-)	−1% (-)	+11% (--)	−41% (+)
M5 average	−11% (--)	−8.3% (--)	−3% (-)	−2% (-)	+18% (--)	−21% (+)
M15-A	−28% (--)	−32% (--)	−6% (-)	−5% (-)	+11% (-)	−16% (+)
M15-C30	−32% (--)	−30% (--)	−12% (-)	−5% (-)	+15% (-)	−39% (+)
M15-C40	−31% (--)	−23% (--)	−12% (-)	−8% (-)	+15% (-)	−43% (+)
M15-S0	−15% (-)	+1% (+)	−3% (-)	−5% (-)	+50% (--)	−20% (+)
M15-S1	−13% (-)	−11% (-)	−4% (-)	−2% (-)	+25% (--)	−40% (+)
M15-S2	−12% (-)	−11% (-)	0%	−2% (-)	+26% (--)	−54% (+)
M15 average	−22% (--)	−18% (--)	−6% (-)	−5% (-)	+24% (--)	−35% (+)
M25-A	−39% (--)	−32% (--)	−1% (-)	−9% (-)	+30% (--)	−54% (+)
M25-C30	−47% (--)	−31% (--)	−20% (-)	−11% (-)	+36% (--)	−55% (+)
M25-C40	−49% (--)	−23% (-)	−19% (-)	−12% (-)	+28% (--)	−50% (+)
M25-S0	−29% (--)	−7% (-)	−8% (-)	−9% (-)	+84% (--)	−21% (+)
M25-S1	−28% (--)	−14% (-)	−5% (-)	−3% (-)	+48% (--)	−39% (+)
M25-S2	−28% (--)	−17% (-)	−8% (-)	−5% (-)	+36% (--)	−60% (+)
M25 average	−37% (--)	−21% (-)	−10% (-)	−8% (-)	+44% (--)	−47% (+)

+: Positive variation with incorporation of MgO in the mortar. -: Negative variation less or equal to the ratio of substitution of cement with MgO. --: Negative variation above the ratio of substitution of cement with MgO.

## Data Availability

No new data were created or analyzed in this study. Data sharing is not applicable to this article.
